# Long term follow-up after haematopoietic stem cell transplantation for mucopolysaccharidosis type I-H: a retrospective study of 51 patients

**DOI:** 10.1038/s41409-022-01886-1

**Published:** 2022-12-09

**Authors:** Antoine Gardin, Martin Castelle, Samia Pichard, Aline Cano, Brigitte Chabrol, Julie Piarroux, Agathe Roubertie, Yann Nadjar, Anne-Sophie Guemann, Marine Tardieu, Didier Lacombe, Matthieu P. Robert, Catherine Caillaud, Roseline Froissart, Virginie Leboeuf, Valérie Barbier, Juliette Bouchereau, Manuel Schiff, Brigitte Fauroux, Briac Thierry, Romain Luscan, Syril James, Timothée de Saint-Denis, Stéphanie Pannier, Cyril Gitiaux, Estelle Vergnaud, Nathalie Boddaert, Claire Lascourreges, Michel Lemoine, Damien Bonnet, Stéphane Blanche, Jean-Hugues Dalle, Bénédicte Neven, Pascale de Lonlay, Anaïs Brassier

**Affiliations:** 1 Department of Pediatric Metabolism, Reference Center of Inherited Metabolic Disorders, Hôpital Necker-Enfants Malades, AP-HP, Université Paris-Cité, Paris, France; 2 Paediatric Hematology Immunology Rheumatology Unit, Hôpital Necker-Enfants Malades, AP-HP, Université Paris-Cité, Paris, France; 3 Department of Neuropediatrics and Metabolism, Reference Center of Inherited Metabolic Disorders, Hôpital Timone Enfants, Marseille, France; 4 Department of Neuropediatrics, Centre Hospitalier Universitaire de Montpellier, Montpellier, France; 5 INM, Univ Montpellier, INSERM U1298, Montpellier, France; 6 Neuro-Metabolism Unit, Reference Center for Lysosomal Diseases, Hôpital Universitaire Pitié-Salpêtrière, AP-HP, Paris, France; 7 Department of Pediatric Metabolism, Reference Center of Inherited Metabolic Disorders, Hôpital Jeanne de Flandre, Lille, France; 8 Department of Pediatrics, Center for Inborn Errors of Metabolism ToTeM, Centre Hospitalier Régional Universitaire de Tours, Tours, France; 9 Department of Medical Genetics, CHU Bordeaux, Université de Bordeaux, INSERM U1211, Bordeaux, France; 10 Department of Ophthalmology, Hôpital Necker-Enfants Malades, AP-HP, Université Paris-Cité, Paris, France; Borelli Centre, UMR 9010 CNRS - SSA - ENS Paris Saclay - Paris Cité University, Paris, France; 11 Biochemistry, Metabolomics, and Proteomics Department, Hôpital Necker-Enfants Malades, AP-HP, Université Paris-Cité, Paris, France; 12 Biochemical and Molecular Biology Department, Lyon University Hospital, Bron, France; 13 Pediatric Noninvasive Ventilation and Sleep Unit, Hôpital Necker-Enfants Malades, AP-HP, Université Paris-Cité, EA 7330 VIFASOM, Paris, France; 14 Department of Pediatric Otolaryngology, Hôpital Necker-Enfants Malades, AP-HP, Université Paris-Cité, Paris, France; 15 Department of Pediatric Neurosurgery, Hôpital Necker-Enfants Malades, AP-HP, Université Paris-Cité, Paris, France; 16 Paediatric Orthopaedic Service, Hôpital Necker-Enfants Malades, AP-HP, Université Paris-Cité, Paris, France; 17 Department of Paediatric Neurophysiology, Hôpital Necker-Enfants Malades, AP-HP, Université Paris-Cité, Paris, France; 18 Department of Anesthesia, Hôpital Necker-Enfants Malades, AP-HP, Université Paris-Cité, Paris, France; 19 Paediatric Radiology Department, AP-HP, Hôpital Necker-Enfants Malades, Université Paris Cité, Institut Imagine INSERM U1163 and U1299, F-75015 Paris, France; 20 Department of Pain and Palliative Care Unit, Hôpital Necker-Enfants Malades, AP-HP, Université Paris-Cité, Paris, France; 21 Department of Physical Medicine and Rehabilitation, Hôpital Necker-Enfants Malades, AP-HP, Université Paris-Cité, Paris, France; 22 Department of Congenital and Pediatric Cardiology, M3C-Necker, Hôpital Necker-Enfants Malades, AP-HP, Université Paris-Cité, Paris, France; 23 Hematology and Immunology Department, Hôpital Robert Debré, GHU AP-HP Nord Université Paris-Cité, Paris, France; 24 Institut Imagine, Paris, France

**Keywords:** Paediatrics, Neurological disorders

## Abstract

Mucopolysaccharidosis type I-H (MPS I-H) is a rare lysosomal storage disorder caused by α-L-Iduronidase deficiency. Early haematopoietic stem cell transplantation (HSCT) is the sole available therapeutic option to preserve neurocognitive functions. We report long-term follow-up (median 9 years, interquartile range 8–16.5) for 51 MPS I-H patients who underwent HSCT between 1986 and 2018 in France. 4 patients died from complications of HSCT and one from disease progression. Complete chimerism and normal α-L-Iduronidase activity were obtained in 84% and 71% of patients respectively. No difference of outcomes was observed between bone marrow and cord blood stem cell sources. All patients acquired independent walking and 91% and 78% acquired intelligible language or reading and writing. Intelligence Quotient evaluation (*n* = 23) showed that 69% had IQ ≥ 70 at last follow-up. 58% of patients had normal or remedial schooling and 62% of the 13 adults had good socio-professional insertion. Skeletal dysplasia as well as vision and hearing impairments progressed despite HSCT, with significant disability. These results provide a long-term assessment of HSCT efficacy in MPS I-H and could be useful in the evaluation of novel promising treatments such as gene therapy.

## Introduction

Mucopolysaccharidosis type I (MPS I) is a lysosomal storage disorder caused by α-L-Iduronidase deficiency, responsible for glycosaminoglycan (GAG) deposition in many tissues. Patients display coarse facial features, hepatosplenomegaly, neurocognitive decline, progressive skeletal dysplasia, heart valvular disease and vision and hearing loss. In its most severe form, Hurler disease (MPS I-H), symptoms are already present before 18 months of age, with death occurring by the age of 10 years in the absence of treatment [1].

Although gene therapy using lentiviral vectors has shown promising results in the treatment of children with MPS I-H [2], haematopoietic stem cell transplantation (HSCT) remains the standard of care to prevent neurocognitive decline, when performed before 24–30 months of age and in the absence of significant pre-existing neurological impairment [3, 4]. The aim of HSCT is to restore a normal enzymatic activity in all organs, and especially in the central nervous system through corrected microglial cells. Due to poor vascularization, corneal clouding and skeletal dysplasia are incompletely corrected following HSCT and they remain the two main disabilities at adulthood despite transplantation [1, 3]. Enzyme replacement therapy (ERT) is the only alternative therapeutic option for MPS I, but it cannot cross the blood-brain barrier and is only effective for soft tissue GAG deposits [5, 6]. Few studies have assessed the long-term outcome after HSCT [3, 7].

In this study, we retrospectively analyzed long-term outcome after HSCT in a multicentric retrospective cohort of MPS I-H patients.

## Material and methods

51 patients with MPS I-H, defined on the severity of the initial clinical presentation, who underwent HSCT between 1986 and 2018 were included in this study. Patients with a Hurler-Scheie (milder) phenotype were excluded. Patients or patients’ parents/legal guardians provided written consent (RADICO National study approved by independent ethics committee). Patients were monitored in reference centers in Paris (*n* = 34), Marseille (*n* = 7), Montpellier (*n* = 5), Lille (*n* = 2), Tours (*n* = 2) or Bordeaux (*n* = 1). Follow-up was performed in their respective centers: closely the first year after HSCT, then every 6 months for check-up and annually for their multidisciplinary evaluation according to published guidelines [8]. Follow-up was more frequent for patients who had resumed ERT after HSCT.

Data from clinical examination, neurocognitive development assessment, ENT or ophtalmologist evaluation, imaging (echocardiography, brain magnetic resonance imaging/MRI), pulmonary function tests and laboratory parameters (whole blood chimerism, urinary GAG quantification, α-L-Iduronidase activity) were retrospectively collected and analyzed. For the neurocognitive evaluation, results of developmental quotient (DQ) before HSCT (Brunet Lezine scale) and intelligence quotient (IQ) after HSCT (WPPSI, WISC or WAIS scales according to age) were graded based on the IQ mental retardation scale: IQ/DQ ≥ 85, between 70 and 85, between 50 and 70 or <50/not evaluable. Academic achievements were classified as follows for children ≥ 6 years of age: 1. Normal or remedial schooling (for minor learning disabilities such as dyslexia, hyperactivity); 2. Specialized schools for intellectual disability; 3. Medically supervised full-time institution (for severe mental retardation). For the adult patients, achievements were graded as follows: 1. Good socio-professional insertion, if patients were able to work or to be involved in vocation training or graduate education; 2. Adapted employment in a specific institution for mentally disabled workers; 3. Neither of the above.

Quantitative variables are presented as median and inter-quartile range Q1–Q3 (IQR) or range (for low sample size). Quantitative variables were compared using the two-sided Student’s *T* test (similar variances) and qualitative variables using the Chi-square test if expected sample sizes were >5 or the Fisher exact test if <5. Survival curves were evaluated using Kaplan–Meier estimates, using the R software (version 4.2.0).

## Results

### HSCT and early outcome

Patients were diagnosed at a median age of 10 months and displayed typical Hurler disease features (Table 1), as well as frequent truncating *IDUA* gene mutations, as described in MPS I-H [9]. Active non-obstructive hydrocephalus was present in 9 patients before HSCT (18%), and half of the patients displayed white matter hyperintensities (WMH) on MRI (Table 1). DQ was higher than 70 in 33 patients (89%) and higher than 85 in 17 patients (46%) (Table 2).

**Table 1:** Pre-HSCT evaluation in patients with mucopolysaccharidosis type I-H.

Age at diagnosis in months (median, IQR), *n* = 49	10 (7–14)	Brain MRI abnormalities (*n*, %)	
Sex ratio F:M, *n* = 51	2: 3	Lateral ventricles enlargement^b^	20/40 (50%)
Clinical features (*n*, %)		WMH or delayed myelination	21/40 (53%)
Typical coarse facial features	47/47 (100%)	Virchow - Robin spaces enlargement	28/35 (80%)
Macrocrania	34/43 (79%)	*IDUA* gene molecular analysis (*n*, %), *n* = 102 alleles	
Thoracolumbar kyphosis	41/44 (93%)	p.Trp402* allele	57/102 (56%)
Airway obstruction^a^	38/41 (93%)	p.Gln70* allele	11/102 (11%)
Umbilical or inguinal hernia	23/41 (56%)	Other nonsense alleles^c^	5/102 (5%)
Hepatomegaly	37/42 (88%)	Indels or splicing alleles^c^	11/102 (11%)
Splenomegaly	17/42 (40%)	Missense alleles^c^	13/102 (12%)
Corneal clouding	29/41 (71%)	Other alleles^c^ (non p.Trp402*/non p.Gln70*)	5/102 (5%)
Active hydrocephalus and/or VP shunt^b^	9/51 (18%)		

*HSCT* Haematopoietic stem cell transplantation, *IQR* Interquartile range, *MRI* Magnetic resonance imaging, *VP* Ventriculoperitoneal shunt, *WMH* White matter hyperintensities.

^a^Airway obstruction defined as chronic rhinopharyngitis or sleep apnea syndrome.

^b^Hydrocephalus is defined as an enlargement of the brain ventricles with signs of transependymal resorption on brain MRI or need for a ventriculoperitoneal shunt. Lateral ventricle enlargement includes patients with active hydrocephalus.

^c^*Nonsense allele* = p.(Arg615*)(*n* = 3), p.(Trp175*)(*n* = 1), p.(Arg628*)(*n* = 1); *Indels/splice alleles* = c.398_403del (*n* = 2), c.46_57del (*n* = 3), c.209del (*n* = 1), c.1695_1705del (*n* = 1), c.580_589 + 36del (*n* = 1), c.972 + 1 G > A (*n* = 1), c.386-2 A > G (*n* = 1), c.50_61del (*n* = 1); *Missense allele* = p.(Pro533Arg)(*n* = 5), p.(Ala327Pro)(*n* = 2), p.(Met504Arg)(*n* = 2), p.(Thr388Arg)(*n* = 1), p.(Met1Leu)(*n* = 1), p.(Ser433Arg)(*n* = 1), p.(Gly51Asp)(*n* = 1); Non p.(Trp402*)/p.(Gln70*): only p.(Trp402*) and p.(Gln70*) have been excluded. Reference sequence: NM_000203.5.

**Table 2:** Haematopoietic stem cell transplantation and long-term outcome.

Age at HSCT in months (median, IQR), *n* = 51	18 (14–25)	Pre-transplant DQ evaluation (Brunet–Lézine scale), *n* = 37		
**Year of HSCT (median, IQR)**, *n* = 51	2009 (2002–2012)	DQ ≥ 85		17/37 (46%)
**HSCT type**^**a**^ **(*n*, %)**, *n* = 45		70 ≤ DQ < 85		16/37 (43%)
Matched sibling donor (MSD)^b^	10/45 (22%)	DQ < 70		4/37 (11%)
Matched unrelated donor (MUD)	20/45 (44%)	**Early outcome (<1 year after HSCT)**, *n* = 51		
Mismatched unrelated donor (MMUD)	14/45 (31%)	Rejection of HSCT (*n*, %)		8/51 (16%)
Others^c^	1/45 (2%)	Acute GVHD^e^		29/49 (59%)
**Stem cell source (*n*, %)**, *n* = 48		Chronic GVHD^f^		7/49 (14%)
Bone marrow	32/48 (67%)	Viral replication^g^		16/49 (33%)
Cord blood unit	16/48 (33%)	Transplant-related mortality (*n*, %)		4/51 (8%)
**HSCT conditioning regimen (*n*, %)**, *n* = 43		**Long-term outcome (≥1 year after HSCT)**, *n* = 47		
Busulfan - fludarabine - ATG	16/43 (37%)	Death from disease progression (*n*, %)		1/47 (2%)
Busulfan - cyclophosphamide - ATG	20/43 (47%)	Follow-up after HSCT in years (median, IQR)		9 (8–16.5)
Busulfan - cyclophosphamide	5/43 (12%)	Chimerism on whole blood^h^	<95% donor	7/45 (16%)
Others^d^	2/43 (4%)		≥95% donor	38/45 (84%)
**Graft CD34**^**+**^ **cell dose (median, IQR)**		α﻿-L-Iduronidase activity^i^	<80% fo control values	12/42 (29%)
Bone marrow (in 10^6^ CD34^+^ per kg), *n* = 20	8 (5.7–17.4)		≥80% of control values	30/42 (71%)
Cord blood unit (in 10^5^ CD34^+^ per kg), *n* = 10	3 (1.8–3.7)	Urinary GAG quantification^j^	Elevated	12/41 (29%)
**Pre-transplant ERT (after 2004)**			Within normal range	29/41 (71%)
Use of pre-transplant ERT (*n*, %)	31/37 (84%)	Substitutive ERT at last follow-up		6/47 (13%)
Duration in weeks (median, IQR), *n* = 31	12 (8.5–18)			

Long-term follow-up was analyzed in patients who survived beyond the first year after transplantation (*n* = 47).

*ATG* Anti-thymocyte globulin, *DQ* Developmental quotient, *ERT* Enzyme replacement therapy, *GAG* Glycosaminoglycans, *GvHD* Graft vs. host disease, *HSCT* Haematopoietic stem cell transplantation, *IQR* Interquartile range

^a^Type of the 2nd HSCT, if initial rejection.

^b^IDUA mutation carrier (*n* = 7), non-carrier (*n* = 1), missing data (*n* = 2).

^c^Mismatched family donor (MMFD, Haploidentical) *n* = 1.

^d^Busulfan – cyclophosphamide – fludarabine – ATG (*n* = 1); Irradiation – cyclophosphamide – fludarabine (*n* = 1).

^e^Cutaneous (*n* = 27/29, 93%), digestive (*n* = 11/29, 38%), hepatic (*n* = 2/29, 7%) with 8 patients (*n* = 8/49, 16%) having GvHD ≥ grade III.

^f^Cutaneous (*n* = 7/7, 100%), digestive (*n* = 4/7, 57%), hepatic (*n* = 3/7, 43%) and/or pulmonary (3/7, 43%) with 3 patients (*n* = 3/49, 6%) having GvHD ≥ grade III.

^g^Viral replication with a need for antiviral or rituximab treatment: EBV (*n* = 11/16, 69%), adenovirus (*n* = 6/16, 38%), CMV (*n* = 2/16, 13%), HHV6 (*n* = 1/16, 6%), HSV encephalitis (*n* = 1/16, 6%).

^h^Mixed chimerism (*n* = 7): median 78%, range 0–92%.

^i^Last value available or before resuming ERT. Exclusion of the two patients continuously treated with ERT since HSCT.

^j^Normal values according to age and laboratory reference ranges.

HSCT was performed at a median age of 18 months (IQR 14–25, Table 2), after 24 months of age in 13 children (median 26 months). Bone marrow was the main stem cell source (67%) whereas all remaining patients received cord blood stem cells. 20 patients (44%) received a graft from matched unrelated donor (MUD), 14 patients (31%) from mismatched unrelated donor (MMUD) and 10 patients (22%) from matched sibling donor (MSD). Among the 10 MSD, 7 were carrier for *IDUA* mutations, 1 was non-carrier and this information was not available for 2 patients. Donor α-L-Iduronidase enzymatic activity was only available for 4 patients with median values of 61% of control (range 49–96%). Conditioning regimen evolved with time, relying on busulfan-cyclophosphamide for HSCT before 2012 or busulfan-fludarabine for more recent HSCT. GvHD prophylaxis included cyclosporine-A (100%), anti-thymocyte globulin (86%), mycophenolate-mofetil (43%), methotrexate (13%) and/or steroids (13%). Since 2004, pre-transplant ERT is available to reduce the storage phenotype while waiting for the HSCT [5, 6], and was given to 31 of the 37 patients transplanted after 2004 (84%). It was stopped at the time of stem cells infusion in 14 patients (45%) or maintained for a median duration of 22 weeks after HSCT (IQR 17–52) for the remaining patients.

8 patients experienced rejection (16%, primary *n* = 3/8, secondary *n* = 5/8), most frequently with cord blood (*n* = 5/8) and a MMUD (*n* = 4/8). One patient did not receive a second HSCT because of parental choice, whereas all 7 remaining patients received a second HSCT after a median delay of 4 months (range 2–27), successful in all. Acute graft vs host disease (GvHD) occurred in 29 patients (59%, Table 2), with 8 patients (16%) having GvHD ≥ grade III, and responded well to treatment in most patients. Chronic GvHD developed in 7 patients (14%), with 3 patients (6%) having GvHD ≥ grade III. Viral replication was the second most frequent complication. 4 patients died from transplant-related mortality (TRM) within the first year after HSCT (severe GvHD: *n* = 2; thrombotic microangiopathy with sepsis: *n* = 1; sepsis few days after HSCT: *n* = 1).

### Long-term follow-up after HSCT

**Fig. 1:**
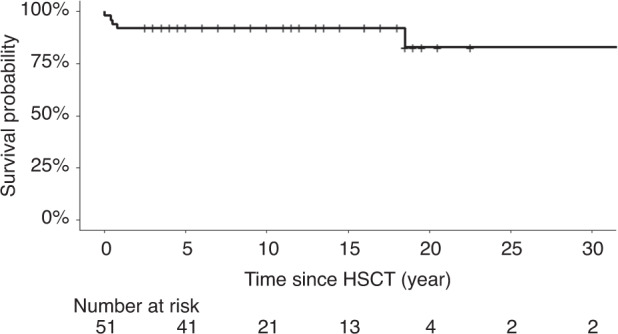
Survival curve of the 51 MPS1-H patients who underwent HSCT. Survival curve made using Kaplan–Meier estimates, using the R software (version 4.2.0).

47 patients survived beyond the first year after HSCT (Fig. 1). The median follow-up after HSCT was 9 years (IQR 8–16.5, Table 2), with 13 patients having reached adulthood (range 18–34 years old). One patient (already reported) died during follow-up at 20 years of age from pulmonary hypertension [10].

At last follow-up, 38 patients out of 45 (84%) displayed a complete chimerism on whole blood (≥ 95% donor chimerism) and 30 patients out of 42 (71%) had leukocyte α-L-Iduronidase activity within normal range (Table 2). None of the patients needed immunosuppressive therapy for GvHD. Serum α-L-Iduronidase activity, available for only 6 patients, was low (median 36% of control values). At last follow-up, 6 patients still received ERT: 2 continuously since HSCT (including the patient with rejection and no second HSCT) and 4 because of disease progression during follow-up (see below).

### Neuro-developmental outcome

All children had a delay in motor milestones and all acquired unaided walking (Table 3). Intelligible speech was acquired by 91% of patients. By the age of 8 years, 78% of patients had acquired writing and reading abilities. 10 patients had hydrocephalus, prior to HSCT in 9 and within a few months after HSCT in a last patient. WMH or cortical atrophy were present in one third of the patients (Table 3). An IQ evaluation was available for 23 patients at a median age of 9 years (IQR 5.7–12) and was within “normal” range (≥70, i.e above -2 standard deviations) in 69% of patients (71% of patients <10 years old and 69% of patients ≥10 years old), with most patients displaying an IQ between 70 and 85 (Table 3). Results were homogenous between “verbal” and “non-verbal” IQ items. There were no signs of neurocognitive regression during follow-up. Academic achievements for patients older than 6 years old (*n* = 43) were comparable to the general population in 25 patients (58%), defined as normal or remedial school (for minor learning disabilities) (Table 3). Nonetheless, 11 patients (26%) required a specialized school for intellectual disability, and 7 patients (16%) required a full-time medically supervised institution. At adulthood (Table 3), 8 out of 13 patients had a good socio-professional insertion, defined as the ability to work (*n* = 3) or to be involved in vocation training (*n* = 4) or graduate education (*n* = 1).

**Table 3:** Long-term “neurocognitive” outcome after HSCT in patients with Mucopolysaccharidosis type I-H.

**Psychomotor development**		50 ≤ IQ < 70	4/23 (17%)
Age at walking in months (median, IQR), *n* = 37	18 (17–24)	IQ < 50 or not evaluable	3/23 (13%)
Age at first sentences in years (median, IQR), *n* = 30	3.5 (3–4)	“Verbal” IQ ≥ 70	15/21 (71%)
Acquisition of an intelligible language	42/46 (91%)	“Non Verbal” IQ ≥ 70	14/21 (67%)
Acquisition of reading/writing at 8 years of age	32/41 (78%)	**Education (for** ≥ **6 years old)**^**b**^, *n* = 43	
**Neurological outcome**		Normal or remedial school	25/43 (58%)
Active hydrocephalus and/or need for a VP shunt	10/47 (21%)	Specialized school (for intellectual disability)	11/43 (26%)
White matter hyperintensities on brain MRI	14/39 (36%)	Medically supervised full-time institution	7/43 (16%)
Cortical atrophy on brain MRI	12/39 (31%)	**Situation in adulthood**^c^, *n* = 13	
**Last IQ evaluation**^**a**^ **(*n*, %)**, *n* = 23		Good socio-professional insertion	8/13 (62%)
IQ ≥ 85	4/23 (17%)	Adapted employment (for mental disability)	3/13 (23%)
70 ≤ IQ < 85	12/23 (52%)	None	2/13 (15%)

Long-term follow-up was analyzed in patients who survived beyond the first year after transplantation (*n* = 47).

*HSCT* Hematopoietic stem cell transplantation, *IQ* Intelligence quotient, *IQR* Interquartile range, *MRI* Magnetic resonance imaging, *VP* Ventriculoperitoneal shunt.

^a^At a median age of 9 years (IQR 5.7–12).

^b^Graded in 3 levels: 1. Normal or remedial schooling (for minor learning disabilities such as dyslexia, hyperactivity); 2. Specialized schools for intellectual disability; 3. Medically supervised full-time institution (for severe mental retardation).

^c^Graded in 3 levels: 1. Good socio-professional insertion, if patients were able to work or to be involved in vocation training or graduate education; 2. Adapted employment in a specific institution for mentally-disabled workers; 3. Neither of the above.

### Non-neurological outcome

Skeletal dysplasia progressed despite HSCT and required surgery for kyphoscoliosis and lower limb deformities in 24 (51%) and 16 patients (34%), respectively (Table 4). Some rarer complications such as thoraco-lumbar spinal cord and C1-C2 cervical cord compressions were respectively observed in 5 and 6 patients, often after 10 years of age. Progression of skeletal dysplasia with age led to a significant motor impairment (use of cane or wheelchair) at last follow-up in 17 patients (37%), including 10 of the 13 adult patients. Seven patients (all adults) completely lost the ability to walk, mainly because of hip dysplasia. Most patients also had short stature, with a median final height of 140 cm (IQR 134–146).

**Table 4:** Long-term “non-neurological” outcome after HSCT in patients with Mucopolysaccharidosis type I-H.

Orthopedic outcome (*n*, %)		Cardiac outcome (*n*, %)	
Surgery for thoraco-lumbar kyphosis	24/47 (51%)	Valvular disease (regurgitation/stenosis)^c^	34/47 (72%)
Surgery for spinal cord compression	5/47 (11%)	Hypertrophic cardiomyopathy	2/47 (4%)
Surgery for cervical cord C1-C2 compression	6/47 (13%)	High blood pressure	4/47 (9%)
Lower limb surgery	16/47 (34%)	**Ophthalmic outcome (*n*, %)**	
Surgery for carpal tunnel syndrome	34/47 (72%)	Corneal clouding	45/47 (96%)
Resurgence of carpal tunnel syndrome after surgery	3/34 (9%)	Retinal dystrophy (cone-rod dystrophy)	5/44 (11%)
**Motor impairment**^**a**^ **(*n*, %)**, *n* = 46		Corneal graft	7/47 (15%)
No functional impairment	15/46 (33%)	Visual acuity (VA) impairment (before corneal graft)^d^	
Fatigability with low walking perimeter	14/46 (30%)	VA ≥ 8/10	15/36 (42%)
Walking with support (cane or intermittent wheelchair)	10/46 (22%)	5/10 < VA < 8/10	4/36 (11%)
Complete loss of walking or permanent wheelchair use	7/46 (15%)	VA ≤ 5/10	17/36 (47%)
**Respiratory-ENT outcome (*n*, %)**		**Others**	
Obstructive sleep apnea (use of non-invasive ventilation)	9/45 (20%)	Height at last follow-up in SD (median, IQR), *n* = 47	−2.6 (−4.4 to −1.3)
Reduction of lung volume in PFT (≤60% of normal)	14/30 (47%)	Final height in cm (median, IQR)^e^, *n* = 16	140 (134–146)
Expected difficulties for tracheal intubation^b^	16/44 (36%)	Recurrence of hernia post-HSCT (*n*, %)	15/45 (33%)
Hearing loss	35/47 (74%)	Surgery for temporo-mandibular joint ankylosis (n, %)	3/24 (13%)

Long-term follow-up was analyzed in patients who survived beyond the first year after transplantation (*n* = 47).

*HSCT* Haematopoietic stem cell transplantation, *IQR* Interquartile range, *PFT* Pulmonary function tests, *SD* Standard deviation, *VA* Visual acuity

^a^Motor impairment was graded in 4 levels using a semi-quantitative scale: 1. *No motor* impairment; 2. *Fatigability* with a short walking distance; 3. *Walking with support* (walking cane or intermittent wheelchair use); 4. *Complete loss of walking* ability or permanent wheelchair use.

^b^Anticipated and/or occurring difficult airway management during anesthesia, defined as requirement for an ENT specialist or optic-fiber intubation.

^c^Mitral (*n* = 33/34, 97%) and/or aortic (*n* = 13/34, 38%), of grade ≤ II in all patients.

^d^Visual acuity of the best eye was used as a readout of the visual impairment.

^e^For patients ≥15 years old.

Sensory impairment was the second main disability, primarily with visual impairment and deafness (Table 4). Almost all patients displayed progressive corneal clouding and a corneal graft was required in 7 patients (15%) at a median age of 20 years, with significant visual improvement. Obvious clinical signs of retinal degeneration (rod-cone dystrophy) were present in 5 patients (11%, median age 20 years). Detailed multimodal imaging (comprising spectral domain optical coherence tomography and ultra-wide field autofluorescence imaging) and electrophysiological assessment (comprising full field electroretinography) were not always systematically performed. Out of 12 patients without clinical signs of retinal involvement who underwent these investigations (median age of 11.5 years old), 6 exhibited signs of retinal dystrophy. Visual acuity was ≤5/10 in half of the patients, including all patients older than 18 years except one. Hearing loss was present in 35 patients (74%) and responsible for speech delay in childhood.

Multiple causes of airway obstruction were present (Table 4). Obstructive sleep apnea (OSA) was common in older patients as already described [11], with 9 patients (20%) requiring non-invasive ventilation in our cohort. 3 patients required surgery for temporo-mandibular ankylosis and maximum mouth opening of less than 1 cm, with disappointing results. 2 patients had typical tracheal involvement with tracheal deformity as well as endoluminal infiltration. Anticipated difficult airway management during general anesthesia was frequent (36%), requiring nasal fiberoptic intubation. Tracheal extubation was often also difficult.

Almost all patients displayed thickened cardiac valves after HSCT, with a valvular stenosis or regurgitation in 34 patients (72%, Table 4). Decreased lung volume due to skeletal dysplasia was present in half of the patients and worsened with age. Nonetheless, no patient had cardio-pulmonary symptoms except the patient who died from pulmonary hypertension [10].

4 patients were treated with ERT during follow-up because of disease progression: one at 16 years of age for severe OSA and mouth opening reduction, despite a 95% donor chimerism and 50% leukocyte α-L-Iduronidase activity; one at 19 years of age due to OSA, severe tracheal stenosis and aortic valve regurgitation with left ventricle dilatation, with a 37% donor chimerism and 60% leukocyte α-L-Iduronidase activity; one at 13 years of age because of OSA and descending aortic stenosis causing upper-body systemic hypertension, despite a full donor chimerism and normal α-L-Iduronidase activity; one at 14 years for airway obstruction and pulmonary hypertension (already reported [10]). There was a striking improvement in soft tissue infiltration with a complete disappearance of OSA, but a lower efficacy for skeletal dysplasia and cardio respiratory features as already reported. No efficacy was reported for the descending aorta stenosis, with the need to implant an aortic stent.

One patient had an uncomplicated and successful spontaneous pregnancy.

### Analysis of outcome according to the stem cell source used

We compared the late outcome as a function of stem cell source among recipients of bone marrow (*n* = 32) and cord blood (*n* = 16) (Table 5). Cord blood transplants were performed more recently and were mainly HLA-mismatched whereas HSCT with bone marrow source were mostly from HLA-matched donors (MSD, MUD). Median follow-up was similar between the two groups, although the IQR range in the bone marrow group was wider. Rates of GvHD, complete chimerism and normal α-L-Iduronidase activity were similar between the two groups. Neurocognitive outcomes were similar between the two groups, whereas there was a trend toward better non-neurological outcomes with cord blood, with higher height at last follow-up (*p* = 0.07) and less valvular disease (*p* = 0.09).

**Table 5:** Comparison of outcomes according to the stem cell source used.

	Bone marrow stem cell source (*n* = 32)	Cord blood stem cell source (*n* = 16)		*p*-value
HSCT features				
Age of HSCT in months (median, IQR)	17 (14.0–25.0)	18 (12.8–22.5)		0.72
Year of HSCT (median, IQR)	2010 (2001–2012)	2012 (2009–2014)		**0.028**
HSCT type^a^ (*n*, %)				
Matched sibling donor (MSD)	9/30 (30%)	1/15 (7%)		
Matched unrelated donor (MUD)	16/30 (53%)	4/15 (27%)		**0.002**^**F**^
Mismatched unrelated donor (MMUD)	4/30 (13%)	10/15 (66%)		
Fludarabine-based conditioning regimen (*n*, %)	14/28 (50%)	4/14 (29%)		0.19
DQ ≥ 70 prior to HSCT (*n*, %)	22/25 (88%)	10/11 (91%)		1.0^F^
Active hydrocephalus or VP shunt pre-HSCT (*n*, %)	7/32 (22%)	1/16 (6%)		0.24^F^
Death (*n*, %)	4/32 (13%)	1/16 (6%)		0.65^F^
Hematological & metabolic outcome				
Acute GvHD (*n*, %)	21/31 (68%)	7/16 (44%)		0.11
Chronic GvHD (*n*, %)	6/31 (19%)	1/16 (6%)		0.40^F^
Complete whole-blood chimerism (≥95% donor), (*n*, %)	24/29 (83%)	13/15 (87%)		1.0^F^
Normal 훂-L-Iduronidase activity (≥80% control values), (*n*, %)	21/28 (75%)	7/12 (58%)		0.45^F^
Normal urinary GAG levels (*n*, %)	19/28 (68%)	8/10 (80%)		0.69^F^
Follow-up post-HSCT in years (median, IQR)	8 (6.8–18.1)	8.0 (6.3–12.2)		0.44
Neurocognitive outcome				
Acquisition of language (*n*, %)	26/29 (90%)	13/14 (93%)		1.0^F^
Aquisition of reading and writing at 8 years of age (*n*, %)	18/25 (72%)	11/13 (85%)		0.46^F^
IQ ≥ 70 at last evaluation (*n*, %)	10/16 (63%)	5/6 (83%)		0.62^F^
Normal or remedial school^b^ (for ≥6 years-old)	15/26 (58%)	8/14 (57%)		0.97
Orthopedic outcome				
Surgery for thoraco-lumbar kyphosis (*n*, %)	16/29 (55%)	8/15 (53%)		0.91
Surgery for cervical cord C1-C2 compression (*n*, %)	5/29 (17%)	1/14 (7%)		0.64^F^
Significant motor impairment^c^ (*n*, %)	11/29 (38%)	5/14 (35%)		0.89
Height at last follow-up in SD (median, IQR)	−3 (−4.9 to −1.3)	−1.7 (−2.8 to −0.6)		0.07
Other non-neurological outcomes				
Obstructive sleep apnea (use of non-invasive ventilation) (*n*, %)	6/29 (21%)	3/13 (23%)		1.0^F^
Valvular disease (regurgitation/stenosis) (*n*, %)	23/29 (79%)	8/15 (53%)		0.09^F^
Visual acuity ≤5/10 (before corneal graft)^d^ (*n*, %)	12/22 (55%)	3/12 (25%)		0.10
Corneal graft (*n*, %)	5/29 (17%)	1/15 (7%)		0.65^F^

HSCT features and outcomes were compared in MPS1-H patients who received HSCT with either bone marrow (*n* = 32) or cord blood (*n* = 16) as a stem cell source. Quantitative variables were compared using Student’s *T* test and qualitative variables using Chi-square test (if expected sample sizes >5) or Fisher exact test (if expected sample sizes <5, labeled with ^F^).

Bold values indicate statistical significance *p* < 0.05.

*DQ* Developmental quotient, *GAG* Glycosaminoglycan, *GvHD* Graft vs host disease, *HSCT* Haematopoietic stem cell transplantation, *IQ* Intelligence quotient, *IQR* Interquartile range, *SD* standard deviation, *VP* Ventriculoperitoneal shunt.

^a^Type of the 2^nd^ HSCT, if initial rejection.

^b^Normal or remedial schooling (for minor learning disabilities such as dyslexia, hyperactivity).

^c^Defined as the requirement of cane or wheelchair for walking.

^d^Visual acuity of the best eye was used as a readout of the visual impairment.

## Discussion

Although HSCT is a validated therapeutic option for patients with MPS I-H, few studies have assessed long-term outcomes [7]. We present herein data on a large cohort of 51 patients with a median follow-up of 9 years after HSCT, including 13 patients having reached adulthood. Although neurocognitive outcome was good in half of the patients, significant disabilities remains, especially because of skeletal dysplasia and visual impairment.

A younger age at HSCT and the absence of a significant developmental delay are known to be associated with a better neurocognitive outcome [1, 3]. DQ is often assessed in France using the Brunet-Lezine scale, but results are often heterogeneous in MPS I-H patients, with better preserved coordination and sociability, and poorer results for postural and language criteria because of hearing loss and skeletal dysplasia [1]. This scale may not reflect their actual cognitive development.

Neuro-developmental outcomes are difficult to assess retrospectively, especially in MPS I-H patients because of confounding factors (hearing loss, visual impairment, motor disabilities). We used semi-quantitative assessments to reflect global neurocognitive outcomes. Neurocognitive outcomes were satisfactory in two-thirds of patients, in line with previous studies [3], and more than half could follow an education in normal or remedial schools. Most patients required a variable degree of assistance in daily activities due to motor and sensory disabilities. Although imperfect, one has to consider the severity of the disease if left untreated [12]. As a comparison, 4 MPS I-H patients could not receive HSCT during the same period (DQ < 70, *n* = 2; no compatible donor, *n* = 1; age >30 months, *n* = 1), and were treated with ERT from a median age of 17 months. After a median follow-up of 10 years, all are severely disabled (unevaluable IQ: *n* = 4/4; no speech: *n* = 2/4; no reading/writing: *n* = 4/4), all require a full-time institution (vs. 16% of HSCT-treated patients) and one died at 9 years of age.

In our study, we did not observe significant differences in cognitive and non-neurological outcomes, chimerism and α-L-Iduronidase enzymatic activity according to the type of stem cell source. While previous studies have suggested that cord blood transplants were associated with better donor chimerism and higher α-L-Iduronidase activity as compared to HSCT performed with bone marrow [13, 14], a recent publication by Orchard et al. showed similar leukocytes and plasma α-L-Iduronidase activity between bone marrow and cord blood HSCT recipients after a longer follow-up [15]. In our study, we did not observe differences in donor chimerism and α-L-Iduronidase enzymatic activity according to the stem cell source, but our cohort was smaller and cord blood was used in only one third of transplants.

As the correction of the neurological features are mediated by corrected microglial cells from the bone marrow and by their secretion of α-L-Iduronidase into the brain [1], it is likely that the patients with a low leukocyte α-L-Iduronidase activity also have a low intra-cerebral activity. A low cerebral α-L-Iduronidase activity despite HSCT was demonstrated in a mouse model of Hurler disease [16]. Serum α-L-Iduronidase activity could better reflect the secretion and enzyme level in tissues. It was only available in 6 patients, and was much lower than the leukocyte enzymatic activity. In addition, a low enzymatic activity could also worsen the hearing loss and vision impairment, as suggested by others [3], and therefore could have an impact on the cognitive outcome.

Hydrocephalus is a known complication of MPS I-H due to cerebrospinal fluid (CSF) resorption impairment, and some studies described that HSCT, but not ERT alone, could prevent its occurrence [3, 12]. Hydrocephalus always occurred prior to HSCT or in the months immediately after HSCT, and with no occurrence during later follow-up, as described [3].

HSCT is unable to fully prevent the development of visceral complications, although milder compared to historical cohorts. In line with previous studies [3, 7], skeletal dysplasia and vision impairment are the two main handicaps in our cohort, likely because of the poor vascularization of bone and cornea. Complex surgeries with prolonged rehabilitation are required, which can significantly impact on neurocognitive development. In older patients as well as in almost all adult patients, a severe reduction of mobility is present, mostly because of hip dysplasia. Finally, height remains low despite HSCT (final height: 140 cm), although it was higher than in previously published cohorts treated with ERT alone (<−3 SD with shorter follow-up [17]), and in our 4 patients without HSCT after a median follow-up of 10 years (median height −4.5 SD, including one adult patient with a final height of 123 cm).

The second most disabling complication is visual impairment. All patients experienced corneal clouding with a progressive decrease of visual acuity and corneal grafts were indicated in the older patients. Retinal dystrophy is present in older patients and can be difficult to assess due to corneal clouding; it is responsible for visual field restriction, hemeralopia, and can eventually worsen visual acuity [18]. In contrast to the hearing loss that persists after HSCT, airway obstruction rapidly improved following ERT and HSCT but can recur post-HSCT, with OSA and anticipated difficult airway management during anesthesia. Some patients resumed ERT with good efficacy, allowing a weaning from non-invasive ventilation. Finally, although most patients displayed reduced lung volume and valvular disease, this was often mild and without clinical symptoms in most of them.

Despite prolonged survival and a favorable neurocognitive outcome, HSCT is still associated with significant morbidity and mortality including rejection and GvHD [13]. 14% of our patients displayed chronic GvHD, which is in line with previous studies (rates ranging from 5.6 to 16%) [13, 14, 19]. Almost all deaths described in our cohort were attributed to TRM, with two patients having experienced fatal chronic GvHD. Nonetheless, no surviving patients still required immunosuppressive therapy at last follow-up. Gene therapy using lentiviral transduction of autologous hematopoietic stem cells could avoid the occurrence of GvHD, reduce the delay between diagnosis and HSCT and allow supraphysiological α-L-Iduronidase activity levels. Studies in mice treated with gene therapy have shown that the level of α-L-Iduronidase in brain is much higher than what can be obtained using HSCT [16]. Recently published results in patients confirmed the safety and the supraphysiological α-L-Iduronidase activity level in both blood and CSF, as well as a striking reduction of GAG accumulation, again both in blood and CSF [2].

Finally, although we did not notice the high rate of severe depression and acute psychotic episode described by others [7], it is clear that the high disease burden remaining after HSCT has a major impact on the quality of life of the patients and could lead to depression.

Our study provides retrospective data on long-term outcome after HSCT in a large cohort of MPS I-H. Currently, HSCT remains the standard of care to both prolong survival, prevent neurological decline and slow the progression of the visceral features of the disease despite severe residual motor and sensory handicaps. Future studies will assess if supraphysiological enzymatic correction obtained using gene therapy will translate in better long-term outcome.

## Data Availability

All data generated or analyzed during this study are included in this published article.
